# Artificial intelligence in communication impacts language and social relationships

**DOI:** 10.1038/s41598-023-30938-9

**Published:** 2023-04-04

**Authors:** Jess Hohenstein, Rene F. Kizilcec, Dominic DiFranzo, Zhila Aghajari, Hannah Mieczkowski, Karen Levy, Mor Naaman, Jeffrey Hancock, Malte F. Jung

**Affiliations:** 1 Department of Information Science, Cornell University, 343 Campus Rd, Ithaca, NY 14853 USA; 2 Department of Computer Science and Engineering, Lehigh University, 113 Research Dr, Bethlehem, PA 18015 USA; 3 Department of Communication, Stanford University, 450 Jane Stanford Way, Stanford, CA 94305 USA; 4 Cornell Tech, 2 W Loop Rd, New York, NY 10044 USA

**Keywords:** Psychology, Engineering

## Abstract

Artificial intelligence (AI) is already widely used in daily communication, but despite concerns about AI’s negative effects on society the social consequences of using it to communicate remain largely unexplored. We investigate the social consequences of one of the most pervasive AI applications, algorithmic response suggestions (“smart replies”), which are used to send billions of messages each day. Two randomized experiments provide evidence that these types of algorithmic recommender systems change how people interact with and perceive one another in both pro-social and anti-social ways. We find that using algorithmic responses changes language and social relationships. More specifically, it increases communication speed, use of positive emotional language, and conversation partners evaluate each other as closer and more cooperative. However, consistent with common assumptions about the adverse effects of AI, people are evaluated more negatively if they are suspected to be using algorithmic responses. Thus, even though AI can increase the speed of communication and improve interpersonal perceptions, the prevailing anti-social connotations of AI undermine these potential benefits if used overtly.

## Introduction

Communication is the basic process through which people form perceptions of others^1^, build and maintain social relationships^2^, and achieve cooperative outcomes^3^. Generative AI that draws from Large Language Models (LLMs) is poised to fundamentally change how we communicate. AI applications like ChatGPT are increasingly used to produce any kind of language, from text messages and social media posts to computer programs and speeches^4–6^.

**Figure 1:**
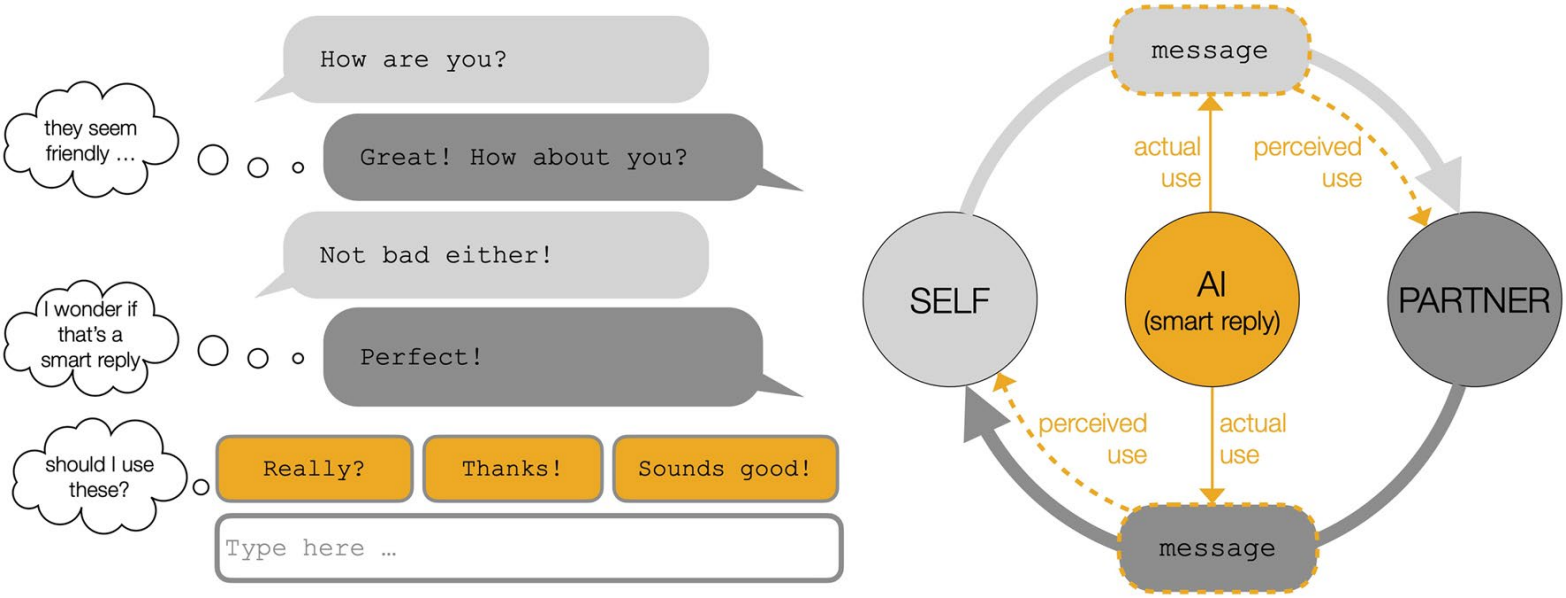
Left side: Example of a message exchange with AI support (i.e. a smart-reply enabled messenger). Typical examples of smart replies and how they might be presented to a user are shown in orange at the bottom. Right side: Abstract representation of the influence of AI on interpersonal communication. Either one or both participants can have access to AI support (e.g. in the form of smart replies). When given access to AI support, participants may choose to use it or not (actual use). However, independent of actual use, participants make assumptions about AI support (perceived use). Both actual use and perceived use influence the overall message exchange and the perceptions people form of each other.

One of the most pervasive AI applications to date is personalized reply suggestions in text-based communication, commonly known as “smart replies”^7^. As of 2017, algorithmic responses constituted 12% of all messages sent through Gmail^8^, representing about 6.7 billion emails written by AI on our behalf each day^9^. Smart reply systems aim to make text production more efficient by drawing on general text corpora to predict what a person might type and generating one or more suggested responses that the person can choose from when responding to a message^7^ (see Fig. 1). Rapid adoption of this type of AI in interpersonal communication has been facilitated by a large body of technical research regarding various methods for generating algorithmic responses^7,10,11^.

Despite the rapid deployment of AI applications in new products and contexts as well as growing concerns about their consequences for society^12^, the scientific community has largely ignored the potential social impacts of integrating AI-generated messages into human communication. Reports from the AI Now Institute liken this scenario to “conducting an experiment without bothering to note the results”^13^ and have repeatedly noted the under-investment in research on the social implications of AI while calling for an increase in interdisciplinary examinations of these systems within human populations^14^.

In response, a growing body of work at the intersection of computer and social sciences is concerned with understanding how AI systems may be influencing human behavior^5,15,16^. Initial studies have found that algorithmic responses can impact how people write^17^, and users perceive that the mere presence of smart replies influences the way that they communicate, in part because of the linguistic skew of smart replies, which tend to express excessive positive emotion as compared to normal conversation^18^. However, we do not know how our social relationships with and perceptions of others are affected when we let algorithms speak on our behalf.

To examine the interpersonal consequences of using AI to generate messages, we developed a custom messaging application and conducted two randomized experiments to study how the display and use of AI-generated smart replies in real-time text-based communication affects how people interact and perceive each other. We show that a widely-deployed smart reply algorithm affects various aspects of interpersonal communication, including communication speed, emotional tone, and interpersonal evaluations in both positive and negative ways.

## Results

### AI Impacts Social Relationships: It is Perceived Negatively but Improves Interpersonal Perceptions

Inspired by theories of how computer-mediated communication can affect intimacy and relationship maintenance^19^, we hypothesized that seeing AI-generated reply suggestions could influence participants’ feelings of connectedness with their conversation partner. To test the effect of AI mediation on interpersonal trait inferences and perceptions of cooperativeness, we developed a novel messaging application (detailed in the Methods section) that allows us not only to control which smart replies are displayed but also to collect data about their use in communication.

To identify the effects and perceptions of algorithmic responses in conversation, we randomly assigned 219 pairs of participants (“self” and “partner”) independently to have smart replies (i.e., suggested responses generated using the Google Reply API^20^) either available to use or not. This resulted in four messaging scenarios: (1) both participants can use smart replies, (2) only the self can use smart replies, (3) only the partner can use smart replies, or (4) neither participant can use smart replies. The availability of smart replies encourages participants to use them in conversation. To estimate the effects of smart reply usage, not its mere availability, on conversation speed, sentiment, and interpersonal outcomes, we use an instrumental variable (IV) approach. IV analysis is an established econometric method to estimate causal effects when the experimental treatment depends on individual adoption^21^. Our instrument is the availability of smart replies for the partner, which is both randomly assigned and unobserved by the self. Participants are also blind to whether any given message they receive is a smart reply. This creates ideal conditions for the exclusion restriction assumption of IV to be satisfied because any effect of the instrument (smart reply availability) on the outcome (e.g., ratings of affiliation) is exclusively through its effect on exposure (proportion of messages from the partner that are smart replies).

Participants engaged in a conversation about a policy issue while our application tracked the presentation and use of smart replies. After completing the conversation, participants were given a definition of smart replies and asked to rate on a scale from 1 (“never”) to 5 (“always”) how often they believed that their partner had used them. They also responded to established survey measures of dominance and affiliation (Revised Interpersonal Adjective Scale^22^). The measure presented participants a list of words that “describe how people interact with other” (e.g. shy, kindhearted, outgoing) and asked them to “rate how accurately each word describes your conversation partner” on a scale from “Extremely inaccurate” (1), to “Extremely accurate” (7). Finally, participants completed a cooperative communication measure^23^ that asked participants to rate their agreement with statements such as “we often criticize each other” on a scale from “Strongly disagree” (1) and “Strongly agree” (7). The presentation of the three post-task measures was randomized between participants to avoid any possible order effects. For detailed information about each measure, please see the supplementary materials.

We find that the availability of algorithmic responses was a strong encouragement to use them in conversation [first-stage: *t*(211) = 13.8, *P*<0.0001]. Smart replies accounted for 14.3% of sent messages on average. Availability of algorithmic responses also resulted in faster communication speed, with 10.2% more messages sent per minute [intent-to-treat estimate: *t*(198) = 2.173, *P* = 0.0309]. Smart replies sped up messaging specifically for the participant who could use them, because the partner’s use of smart replies did not significantly improve communication speed of the self [IV estimate: *b* = 0.402, *t*(205) = 0.825, *P* = 0.410]. While smart replies can improve communication speed, their consequences for interpersonal perceptions are more complex.

**Figure 2:**
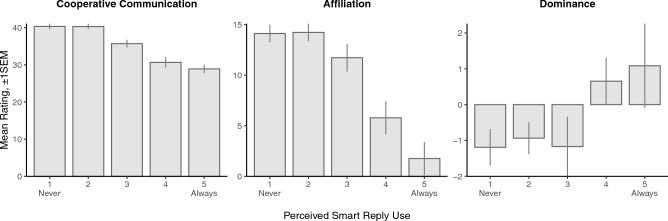
Average rating of the partner’s cooperative communication, affiliation, and dominance by the self for different levels of perceived smart reply (SR) use by the partner (*N* = 361). Error bars show one cluster-robust standard error above and below the mean. See Supplementary Table S6 for details about the frequency of responses per response category.

Participants are capable of recognizing their partner’s use of smart replies to some degree: beliefs about how much their partner used smart replies correlated with actual use but not strongly [Pearson’s *r* = 0.22, *t*(97) = 3.62, *P* = 0.0005]. Consistent with commonly held beliefs about the negative implications of AI in social interactions^24,25^, we find strong associations between perceived smart reply use by the partner and attitudes towards them. The more participants thought their partner used smart replies, the less cooperative they rated them [*t*(92) = −9.89, *P* < 0.0001], the less affiliation they felt towards them [*t*(92) = −6.90, *P* < 0.0001], and the more dominant they rated them [*t*(92) = 2.27, *P* = 0.0256], as shown in Fig. 2, even after controlling for their partner’s actual smart reply use. This shows correlationally that people who appear to be using smart replies in conversation pay an interpersonal toll, even if they are not actually using smart replies. However, this finding does not show causally how attitudes shift in response to actual smart reply use.

We find that increased use of smart replies by the partner actually improved the self’s rating of the partner’s cooperation [IV estimate: *b* = 15.66, *t*(189) = 2.39, *P* = 0.018] and sense of affiliation towards them [IV estimate: *b* = 21.79, *t*(189) = 2.75, *P* = 0.007], but not dominance [IV estimate: *b* = −0.53, *t*(189) = −0.13, *P* = 0.90]. Although perceived smart reply use is judged negatively, actual use by the partner resulted in more positive attitudes. Notably, ratings of cooperation and affiliation were not significantly affected by the presence of algorithmic responses for the self [intent-to-treat estimates: cooperation *b* = 0.397, *t*(188) = 0.436, *P* = 0.663; affiliation *b* = −0.397, *t*(188) = −0.362, *P* = 0.718], only ratings of dominance were reduced given the presence of algorithmic responses for the self [*b* = −1.338, *t*(188) = −2.233, *P* = 0.021].

We also find that increased use of smart replies by the partner led the self to send messages with more positive sentiment [IV estimate: *b* = 0.178, *t*(205) = 2.02, *P* = 0.045], even if smart reply messages were excluded from the sentiment score [*b* = 0.208, *t*(205) = 2.17, *P* = 0.031]. The self’s message sentiment was also more positive if algorithmic responses were available to the self [intent-to-treat estimate: *b* = 0.026, *t*(198) = 2.05, *P* = 0.0422], unless the calculation of message sentiment omits smart reply messages [*b* = 0.019, *t*(198) = 1.35, *P* = 0.1801]. This suggests that merely showing algorithmic responses did not affect the sentiment of written messages, but rather, it affected message sentiment by using smart reply messages which tend to have positive sentiment. Taken together, these findings imply that the effects of AI mediation on interpersonal perceptions are related to changes in language introduced by the AI system.

### AI impacts language: its sentiment affects emotional content in human conversations

To better understand how the sentiment of AI-suggested responses affects conversational language, we conducted a second experiment. Using a between-subjects design, we randomly assigned 291 pairs to discuss a policy issue using our app in one of four conditions: (1) Google smart replies (generated using the Google Reply API^20^), (2) positive smart replies (rated by crowdworkers to have positive sentiment), (3) negative smart replies (rated by crowdworkers to have negative sentiment), or (4) no smart replies were made available to both participants to use in conversation. We measured conversation sentiment using VADER, a lexicon- and rule-based sentiment analysis tool that is ideal for analyzing short, social messages^26^. As a precursor to the VADER score analysis, we used the LIWC affect dictionary^27^ to confirm that smart replies introduced more affective language into the conversation (see Methods section). We aggregated VADER scores into a sentiment polarity score ranking from most positive (1) to most negative (−1), with neutral (0) in the middle. On average, conversations lasted for 6.33 min [*SD*=2.67] and used 20 messages including smart replies.

**Figure 3:**
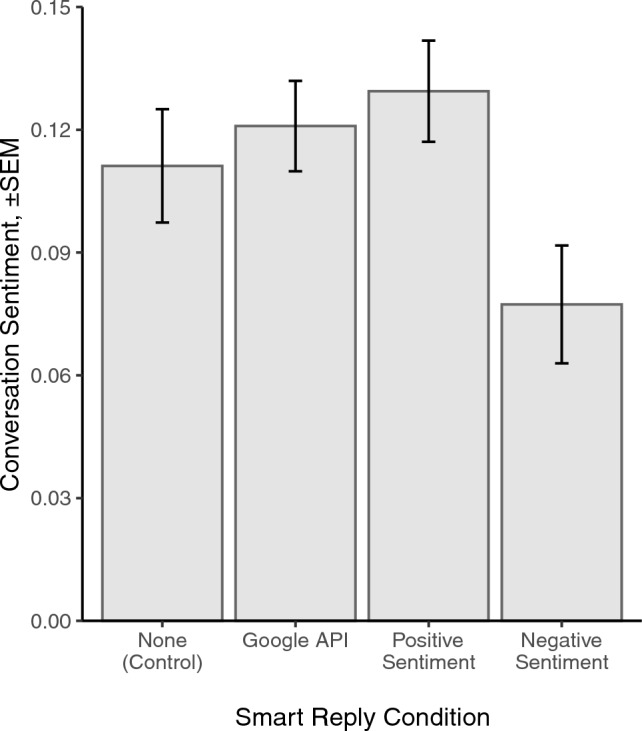
Mean overall conversation sentiment by experimental condition: both participants assigned to no smart replies, negative, positive, or Google smart replies. Error bars show one cluster-robust standard error above and below the mean.

We find that the availability of negative smart replies caused conversations to have more negative emotional content than conversations with positive smart replies [*t*(127) = 2.75, *P* = 0.007, *d* = .352] and the widely-used Google smart replies [*t*(127)=2.40, *P* = 0.018, *d* = .323; Fig. 3], which highlights the positive sentiment bias of smart replies in commercial messaging apps. Google smart replies had a similar effect on conversation sentiment as a set of positive smart replies [*t*(150) = 0.51, *P* = 0.61], but did not cause significantly more positive sentiment compared to having no smart replies available [*t*(137) = 0.55, *P* = 0.58]. Moreover, we find that these shifts in language are driven by people’s *use* of smart replies rather than mere *exposure* to smart reply suggestions; repeating the analysis with smart reply messages omitted from the conversation corpus, we find minimal differences in conversation sentiment between the smart reply conditions [*F*(3277) = 0.360, *P* = 0.782]. Taken together, these findings demonstrate how AI-generated sentiment affects the emotional language used in human conversation.

## Discussion

Our research shows that generative AI, including a commercially-deployed AI system, can have a significant impact on how people communicate with both positive and negative consequences. We find that people choose to use AI when given the opportunity, and this increases the speed of communication and leads to more emotionally positive language. However, we also find that when participants *think* that their partner is using more algorithmic responses, they perceive them as less cooperative, less affiliative and more dominant. This finding could be related to common assumptions about the negative implications of AI in social interactions. For example, humans are already predisposed to trust other humans over computers^25^, and most current communication systems featuring AI mediation lack transparency for users (i.e., the sender knows that their responses have been modified or generated by AI, while the receiver does not). Taken together with users’ preference for reducing uncertainty in interactions^28^, this could lead to negative perceptions of AI in everyday communication. Indeed, these negative perceptions confirm recent findings that people believe that smart replies often do not capture what they want to say and could alter the way that they communicate with others^18^, and that text *suspected* of or labeled as generated by an AI was perceived as less trustworthy^24^.

Despite these negative perceptions of AI in communication, we find that as people *actually* use more algorithmic responses, their communication partner has more positive attitudes about them. Even though *perceived* smart reply use is viewed negatively, *actual* smart reply use results in communicators being viewed as being more cooperative and affiliative. In other words, it seems that the negative perception of using AI to help us communicate does not match the reality.

It is important to note that these findings are specifically related to *using* AI in communication and are not observable when we consider instances where users are simply *presented* with AI recommendations but do not use them. In other words, although we did not find any main effects of being exposed to smart replies, we instead find that the presentation of smart replies acts as an encouragement to use them, and by using them, people are tweaking their language and the way that they are perceived by others.

Our work has implications for theory in communication and psychology. We provide evidence that using AI can shape language production and associated interpersonal perceptions. Understanding this impact is important because language is inextricably linked with listeners’ characterizations of a communicator, including their personality^1^, emotions^2^, sentiment^26,29^, and level of dominance^30^. Indeed, we find that using AI-generated responses changed the expression of emotion in human conversations. The influence of AI on human emotional communication is deeply concerning given that AI is writing billions of emails for us every day^9^. With the increasing popularity of other forms of AI mediating our everyday communication (e.g., Smart Compose^31^), we have little insight into how regularly people are allowing AI to help them communicate or the potential long-term implications of the interference of AI in human communication. Our work suggests that interpersonal relationships are likely to be affected, potentially positively, but future research needs to investigate the longitudinal effects of such changes. For example, could this tweaking of our language potentially lead to a loss of personal communication style, with language expression becoming increasingly homogeneous over time?

This work also has implications for research in computer science that focuses on AI development, as we highlight both opportunities and risks of deploying such systems. We demonstrate how AI systems can influence interactions in positive ways through exceedingly subtle forms of intervention. Merely providing reply suggestions can change the language used in a conversation, with changes being consistent with the linguistic qualities of the algorithmic responses. Additionally, previous work has shown that when conversations go awry, people trust the AI more than their communication partner and assign some of the blame that they otherwise would have assigned to this person to the AI^32^. Taken together, these findings suggest possible opportunities for developers to affect conversational dynamics and outcomes by carefully controlling the linguistics of smart replies that are shown to people^33^. However, this also raises potential risks as AI continues to become increasingly present in our social interactions. With this knowledge, it is important for researchers and practitioners to consider the broader social consequences when designing algorithms that support communication.

Overall, we show how an AI system designed to help people can have unintended social consequences. AI has the potential to help people communicate more quickly and improve interpersonal perceptions in everyday conversation, but our findings caution that these benefits are coupled with alterations to the emotional aspects of our language, and we do not know the effects that such changes could have on communication patterns over time.

## Methods

All methods were carried out in accordance with relevant ethics guidelines and regulations. All experimental protocols and materials were approved by Cornell University’s Institutional Review Board for Human Participant Research (IRB) (Protocol Number: 1610006732): https://researchservices.cornell.edu. Informed consent was obtained from all participants and study 1 was pre-registered on AsPredicted^34^.

### Study 1

We randomly assigned pairs of participants (“self” and “partner”) independently to have smart replies either available to use or not while engaged in a conversation about a policy issue. This resulted in four conditions: (1) both participants can use smart replies, (2) only the self can use smart replies, (3) only the partner can use smart replies, or (4) neither participant can use smart replies. Inspired by theories of how computer-mediated communication can affect intimacy and relationship maintenance^19^, we expected that seeing AI-generated reply suggestions would influence participants’ perceptions of their conversation partner as well as their language.

#### Participants

We recruited 438 Mechanical Turk crowdworkers to this study in return for monetary compensation. Research has shown that data provided by MTurk participants often meets or even exceeds “the psychometric standards set by data collected using other means”^35^. The sample size is comparable to recent other studies that examined the social consequences of algorithmically mediated communication^32,36^.

Because the focus of our research is on full conversations, we excluded conversations with less than 10 messages exchanged overall and those during which a single participant sent less than 3 messages (one pair of participants). We additionally exclude six pairs of participants who did not engage in a meaningful conversation and instead primarily clicked the smart replies (over 75% of messages sent are smart replies). This results in 424 participants for analyses focused on smart reply use. Conversations lasted for 6.81 min on average (*SD* = 2.31) and comprised 21.0 messages on average (*SD* = 7.55). For the analysis of post-conversation self-report outcomes, we also excluded participants who did not complete the full survey (63 participants). This left *N* = 361 (124 women, 235 men, 1 other gender) for survey-based analyses. Participants ranged in age from 18 to 68 (*M* = 34.07, *SD* = 10.1).

#### Smart reply research platform

We developed a flexible web-based research tool called Moshi, that allowed us to recruit participants online and engage them in real-time interpersonal communication tasks while receiving smart reply support.

Moshi is designed as a web application that allows two participants to text chat with one another. Like in existing commercial messaging applications that feature smart replies, participants can also be presented with smart replies that they can tap to send in addition to the standard text box for typing messages. This research tool, available for use by others (https://github.com/Social-Design-Lab/moshi), provides researchers with an experimental platform giving them full control over the type of smart replies that are displayed, how and when they are displayed and who sees them (please see the Supplementary file for more details).

We developed two messenger modes for study 1: No smart replies and real smart replies. Each mode could be activated independently for a participant. In the no smart reply mode, participants had to manually type each message that they sent. The real smart reply mode uses Google’s Reply model^20^ to generate smart replies.

#### Measures

To assess the impact of smart replies on social relationships, we measured perceived dominance and affiliation, and perceived cooperative communication toward the respective conversation partner as well as perceived smart reply use. To assess the impact of smart reply on language we measured communication speed, and messaging sentiment.

*Perceived dominance and affiliation* were operationalized through the revised interpersonal adjective scales (IAS-R). The IAS-R provides an empirical measure of various dimensions that underlie interpersonal transactions^22^. To shorten the measure, two adjectives with the highest loading factors from each interpersonal octant were selected, based on the analysis of Wiggins and colleagues^22^, resulting in 16 items to be ranked. The instructions read, “Below are a list of words that describe how people interact with others. Based on your intuition, please rate how accurately each word describes your conversation partner” (adapted from^37^). Participants rated each statement on rating-scale items anchored by “Extremely inaccurate” (1), “Somewhat accurate” (4), and “Extremely accurate” (7). These ratings were then combined according to a formula adapted from^22^ to determine ratings of affiliation and dominance^37^ (See Appendix for details).

*Perceived cooperative communication* was operationalized through a 7-item scale^23^ where participants rated their agreement with statements describing cooperative communication in their overall interaction with their partner. The instructions read, “Thinking about your interaction with your partner, please rate the extent to which you agree with each of these statements.” Participants rated each statement on rating-scale items anchored by “Strongly disagree” (1) and “Strongly agree” (7).

*Perceived smart reply use* was operationalized by asking participants how often they believed their partner used smart replies on a 5-point scale ranging from “1= Never” to “5 = Always”. The presentation of all post-task survey measures was randomized between participants to address potential order effects in responses.

*Communication speed* was operationalized by calculating the average number of messages a participant sent per minute.

*Messaging sentiment* was operationalized using VADER, a lexicon and rule-based sentiment analysis tool specifically attuned to sentiments expressed on social media^26^. This analysis tool yields a sentiment metric indicating how positive, negative, or neutral the sentiment of the supplied text is. For our purposes, messages were analyzed individually using the VADER compound sentiment output, an aggregated score ranging from −1 to 1 (i.e., most negative to most positive) based on the three aforementioned sentiment components.

#### Procedure

Participants were directed to a Qualtrics survey that guided them through the study procedure. After obtaining informed consent, participants were informed that they would be using a messaging system to complete a discussion task with an anonymous partner. Participants were then presented with a task involving a discussion of unfair rejection of work, an issue that is relevant to crowdworkers^38^. Specifically, we asked pairs to come to an agreement on the “top 3 changes that Mechanical Turk could make to better handle unfairly rejected work.” After opening the messaging platform, participants waited up to 5 min for another participant to enter the conversation. If 5 min elapsed without another participant arriving, participants were able to prematurely exit the survey and receive partial compensation. Once another participant arrived, the pair had as much time as they needed to come to an agreement on a ranked list. After verifying that a conversation was completed, participants were directed to our post-task measures.

#### Data analysis

Following standard procedure for Instrumental Variable (IV) estimation, we compute three types of estimands: first-stage effects, intent-to-treat effects, and IV effects ^21^. In all cases, we compute cluster-robust standard errors (i.e., CR2) using the *coef_test* function in the clubSandwich R package^39^. The first-stage effects estimate how much random assignment to smart reply availability led participants to use smart replies in conversation. The intent-to-treat effects estimate how much assignment to smart reply availability caused changes in outcome measures, such as ratings on the post-survey, communication speed or sentiment. The IV effects estimate the marginal effects of increased smart reply use by the partner on outcomes for the self. Specifically, we analyzed outcome data for the self using IV regression with partner smart reply use instrumented by partner random assignment to condition; the self’s randomly assigned condition was added as a covariate. We used the *ivreg* function in the AER R package^40^. The reported estimates represent coefficients, t-statistics and *p*-values from the IV regression output.

We use an IV approach to estimate the effects of smart reply use (instrumented by randomly assigned availability) on conversation speed, sentiment, and interpersonal perceptions (dominance, affiliation, and cooperative communication). The exclusion restriction assumption is plausible by virtue of the experimental design, because neither participant is informed about their partner’s smart reply availability or whether any given message is a smart reply.

### Study 2

To better understand how the sentiment of AI-suggested responses affects conversational language, we conducted a second experiment. Using a between-subjects design, we randomly assigned 291 pairs to discuss a policy issue using our app in one of four conditions: (1) Both participants receive Google smart replies, (2) both participants receive smart replies with positive sentiment), (3) both participants receive negative smart replies with negative sentiment, or (4) no smart replies.

#### Participants

Across all conditions, 582 Mechanical Turk crowdworkers participated in this study and received monetary compensation for their time. We excluded 13 pairs of participants with less than 10 messages exchanged overall and where one participant sent less than 3 messages. Conversations lasted for 6.33 min on average (*SD* = 2.67) and consisted of 20.2 messages on average (*SD* = 8.63). From a brief post-conversation survey, completed by 510 participants (92%), we know that participants ranged in age from 19 to 69 (*M* = 35.6, *SD* = 9.97), 206 women, 275 men, and one other gender.

#### Materials and measures

We used the same research platform as in study 1 but extended it with two additional modes: Positive and negative sentiment smart replies. For example, in the positive smart reply condition, a participant might see smart replies such as, “I like it” and “I can’t agree more”, whereas in the negative smart reply condition, a participant might see smart replies such as, “I don’t get it” and “No you are not”. These smart replies were chosen randomly from an input file without being too repetitive (i.e., all three utterances shown in each instance are different, and the same utterance is not shown in immediately subsequent instances). Utterances were chosen from previous work^18^ that asked crowdworkers to rate the sentiment of smart replies. Smart reply suggestions included only those that were rated as having definitive positive or negative sentiment, respectively.

To assess the impact of smart replies on language, we measured messaging sentiment. The measure was operationalized as in study 1.

#### Procedure

Procedures were similar to study 1, except participants in the smart reply conditions were informed that they would be “[...] using an AI-mediated messaging system to have a conversation with your partner. While you are messaging, artificial intelligence (AI) will provide smart replies that you can simply tap to send.”, while participants in the control condition were told that they would be “[...] using a standard messaging system to have a conversation with your partner”.

#### Data analysis

We analyzed the resulting data at the individual level using a simple linear regression with cluster-robust standard errors using the *lm_robust* function in the estimatr R package^41^. The dependent variable was the individual language measure (i.e., VADER sentiment) and the independent variable was the assigned condition; no covariates were added. The reported statistics are the t-statistic and *p*-value for the relevant coefficient, and Cohen’s d computed manually.

To ensure that any language differences that we found were not the result of demographic differences between the four conditions^42^, we examined the demographic makeup (i.e., age, gender, and race) between conditions and did not find any significant differences.

As a precursor to the VADER sentiment analysis, we examined the *affect* measure provided by the Linguistic Inquiry and Word Count (LIWC), a dictionary-based text analysis tool that determines the percentage of words that reflect a number of linguistic processes, psychological processes, and personal concerns^26,27^. We use the LIWC Affect score to check if the use of affective language changes with the introduction and use of smart replies. Affect, with values ranging from 0 to 100, is operationalized as the sum of the Positive Emotion and Negative Emotion scores in LIWC.

We found that the presence of positive and Google smart replies caused conversations to have higher affect than conversations without smart replies (*t*(124) = 2.95, *P* <  0.001, *d* = 0.272). The effect of positive and Google smart replies on affect was statistically similar (*t*(150) = 0.354, *P* = 0.724). The presence of negative smart replies had a strong negative effect on conversation affect compared to the control condition without smart replies (*t*(123) = −3.50, *P* <  0.001, *d* = 0.454). Taken together, these findings demonstrate how AI-generated sentiment affects the emotional language used in human conversation.

### Supplementary Information

Supplementary Information.

## Data Availability

The datasets generated and analyzed during the current studies are available in a Mendeley repository^43^, http://dx.doi.org/10.17632/6v5r6jmd3y.1. Due to the potentially sensitive nature of information revealed by participants in the conversations, participants were assured that the raw conversation data would remain confidential and not be shared.
