# Publisher Correction: Artificial intelligence in communication impacts language and social relationships

**DOI:** 10.1038/s41598-023-43601-0

**Published:** 2023-10-03

**Authors:** Jess Hohenstein, Rene F. Kizilcec, Dominic DiFranzo, Zhila Aghajari, Hannah Mieczkowski, Karen Levy, Mor Naaman, Jeffrey Hancock, Malte F. Jung

**Affiliations:** 1 Department of Information Science, Cornell University, 343 Campus Rd, Ithaca, NY 14853 USA; 2 Department of Computer Science and Engineering, Lehigh University, 113 Research Dr, Bethlehem, PA 18015 USA; 3 Department of Communication, Stanford University, 450 Jane Stanford Way, Stanford, CA 94305 USA; 4 Cornell Tech, 2 W Loop Rd, New York, NY 10044 USA

Correction to: *Scientific Reports* 10.1038/s41598-023-30938-9, published online 04 April 2023

The Funding section in the original version of this Article was omitted. The Funding section now reads:

"This material is based upon work supported by the National Science Foundation under Grant No. CHS 1901151."

The original Article has been corrected.

