# Supplementary material for: Artificial intelligence in communication impacts language and social relationships
Source: Sci Rep. 2023 Apr 4;13:5487. doi: 10.1038/s41598-023-30938-9 (PMC10073210; doi:10.1038/s41598-023-30938-9)
Supplement: Supplementary file 1 — Supplementary Information. [file 41598_2023_30938_MOESM1_ESM.pdf]

# Supplementary Materials

## Smart Reply Research Platform

Moshi is a web-based research platform that enables researchers to engage online participants in text-based, real-time interpersonal communication. Moshi takes its design inspiration from commercial text-based AI-MC platforms like Google's since-deprecated platform Allo which has been used to study text-based AI-MC in previous studies \cite{hohenstein2018ai,hohenstein2020ai}. “Allo” means hello in French, and, similarly, we named our platform Moshi, as “Moshi Moshi” means hello in Japanese when answering the phone.

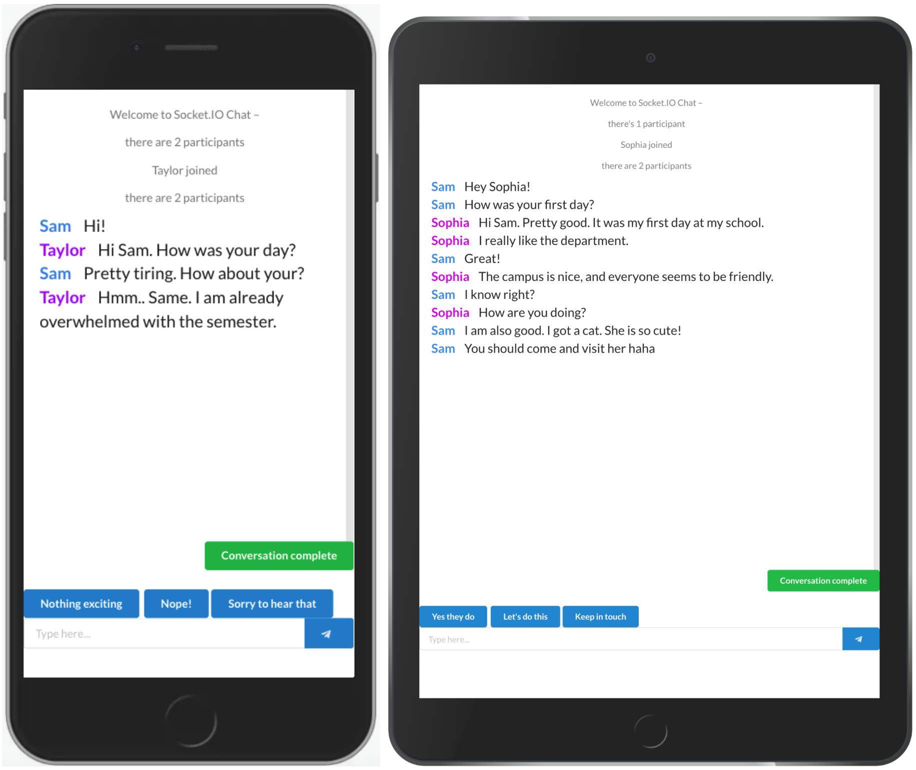


*Figure S1: Example screens of the research platform from the perspective of a study participant. Smart replies are displayed in blue.*

Moshi is a web application that runs on all major modern browsers (i.e., Google Chrome, Mozilla Firefox, Microsoft Edge and Apple Safari) and does not require participants to install anything. The interface is reactive to device type and resizes itself to work well on desktops, tablets, and mobile devices (e.g., Android and iOS).

In addition to the standard text box to send messages, participants can also receive smart replies that they can click or tap to send automatically. Similar to existing chat apps, users can also scroll to see the history of the conversation at any point.

Moshi provides a modular scaffold for experiments on text based AI-MC, allowing other researchers to modify it and explore their own research questions. Moshi allows researchers to define and build their study, the look and feel of the platform, the number of participants in a room, etc. Once set up, researchers need only to point participants to their Moshi instance. Moshi automatically assigns and connects participants into private rooms, measure the study time, collect behavioral data (i.e. the text the enter, buttons they press, the timing of these events, etc.)

Moshi can be downloaded here: [https://github.com/Social-Design-Lab/moshi](https://github.com/Social-Design-Lab/moshi)

# Measures

## 1. Perceived Smart Reply Use

Participants were provided with the following definition and example of smart replies before being asked to rate their interaction partner's perceived smart reply use:

> *Smart replies are messages generated by artificial intelligence (AI) that you can simply tap to send, such as shown below:*

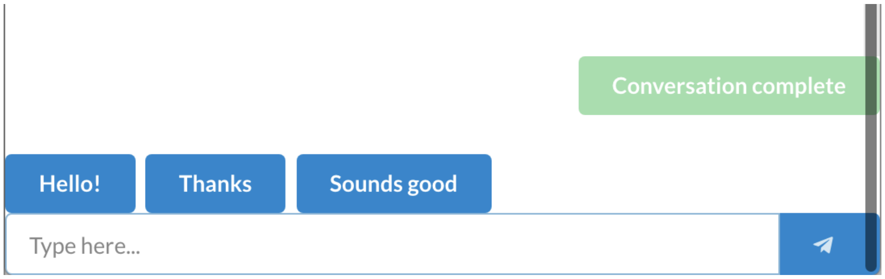


*Figure S2: Screenshot showing how smart replies are displayed as part of the messenger.*

We then used a single item to measure perceived smart reply use:

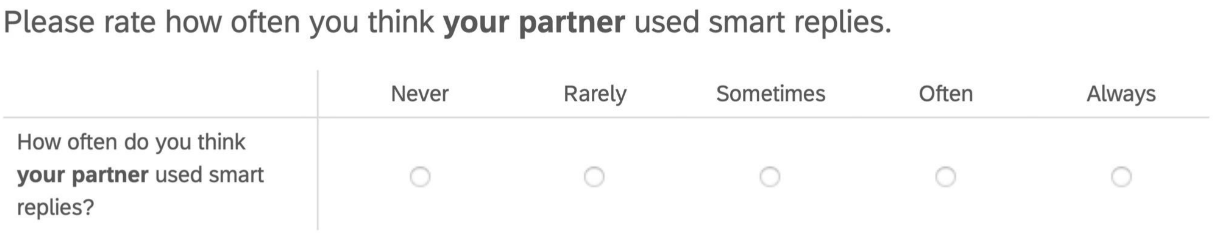


*Figure S3: Perceived smart reply use measure*

## 2. Dominance and Affiliation

We used the Revised Interpersonal Adjective Scales (IAS-R) (Wiggins, Trapnell, & Phillips, 1988) to measure dominance and affiliation. The IAS-R provides an empirical measure of various dimensions that underlie interpersonal transactions. To shorten the measure, two adjectives with the highest loading factors from each interpersonal octant were selected, based on the analysis of Wiggins et al (1988), resulting in 16 items to be ranked. The instructions read, "Below are a list of words that describe how people interact with others. Based on your intuition, please rate how accurately each word describes your conversation partner" (adapted from Knutson, 1996). Participants rated each statement on rating-scale items anchored by

"Extremely inaccurate" (1), "Somewhat accurate" (4), and "Extremely accurate" (7). These ratings were then combined according to a formula adapted from (Wiggins, et al.,  1988) to determine ratings of affiliation and dominance (Knutson, 1996).

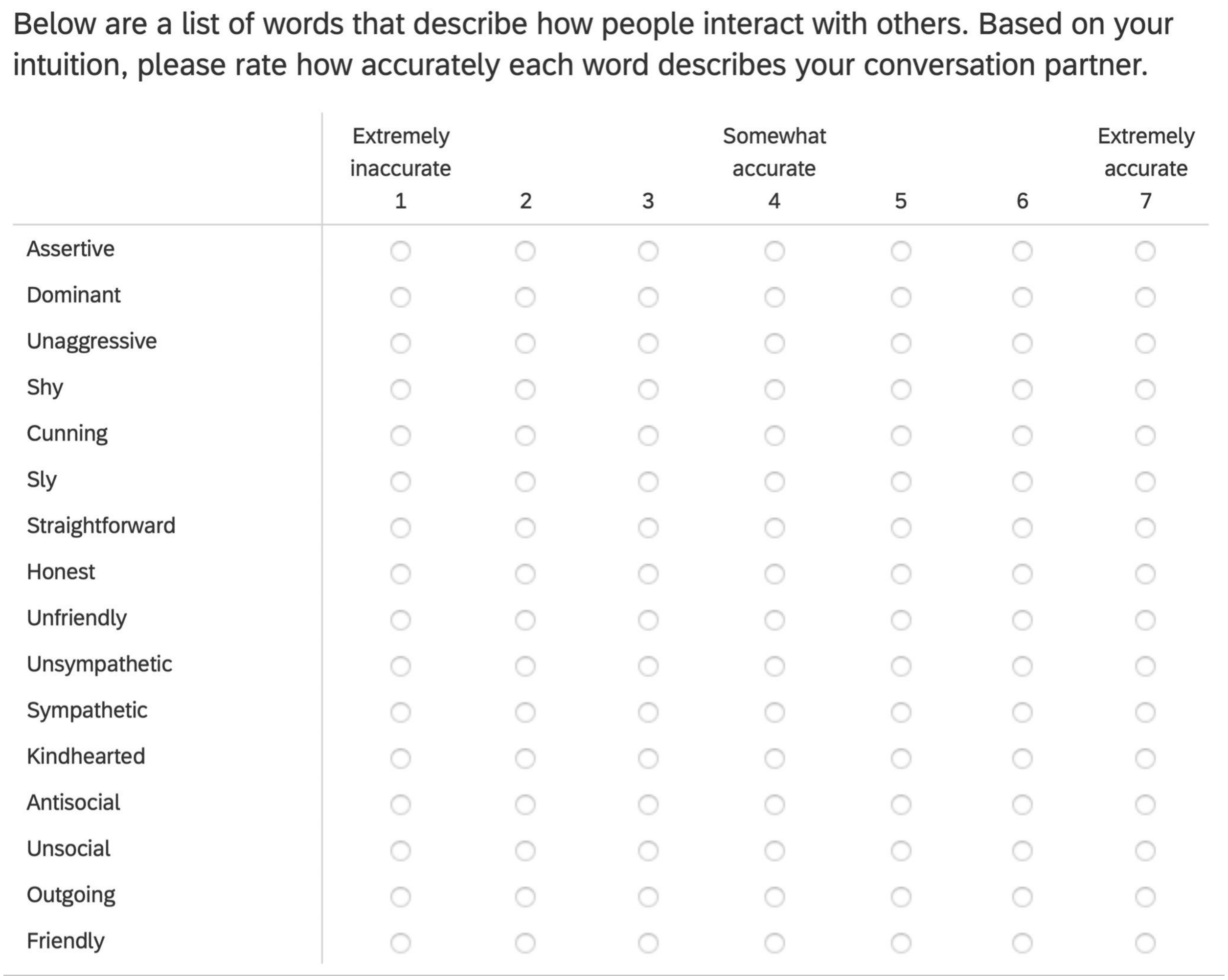
Below are a list of words that describe how people interact with others. Based on your intuition, please rate how accurately each word describes your conversation partner.

| | Extremely inaccurate 1 | 2 | 3 | Somewhat accurate 4 | 5 | 6 | Extremely accurate 7 |
|---|---|---|---|---|---|---|---|
| Assertive | | | | | | | |
| Dominant | | | | | | | |
| Unaggressive | | | | | | | |
| Shy | | | | | | | |
| Cunning | | | | | | | |
| Sly | | | | | | | |
| Straightforward | | | | | | | |
| Honest | | | | | | | |
| Unfriendly | | | | | | | |
| Unsympathetic | | | | | | | |
| Sympathetic | | | | | | | |
| Kindhearted | | | | | | | |
| Antisocial | | | | | | | |
| Unsocial | | | | | | | |
| Outgoing | | | | | | | |
| Friendly | | | | | | | |

*Figure S4: Survey items for the measurement of affiliation and dominance.*

We then used the following procedure for deriving aggregate measures of dominance and affiliation from the IAS-R (adapted from (Wiggins, Trapnell, & Phillips, 1988; Knutson, 1996)):

1. Octant scores are computed from adjective ratings:

PA = (dominant + assertive)/2
BC = (sly + cunning)/2
DE = (unsympathetic + warmthless)/2
FG = (unsociable + antisocial)/2
H1 = (shy + unaggressive)/2
JK = (uncunning + unsly)/2
LM = (gentle + tender)/2

NO = (friendly + outgoing)/2

2. Dominance (DOM) and affiliation (AFF) scores are computed from these octant scores:

DOM = PA - HI + .707(NO + BC - FG - JK)
AFF = LM - DE + .707(NO - BC - FG + JK)

## 3. Perceived Cooperative Communication

We measured the degree of perceived cooperative communication by slightly adapting the cooperative communication measure by Lee (1997) to fit the context of our chat interactions:

Thinking about your interaction with your partner, please rate the extent to which you agree with each of these statements.

| | Strongly disagree | Disagree | Somewhat disagree | Neither agree nor disagree | Somewhat agree | Agree | Strongly agree |
|---|---|---|---|---|---|---|---|
| We openly exchanged relevant information. | ○ | ○ | ○ | ○ | ○ | ○ | ○ |
| We often criticized each other. | ○ | ○ | ○ | ○ | ○ | ○ | ○ |
| We intentionally provided misleading information to each other. | ○ | ○ | ○ | ○ | ○ | ○ | ○ |
| If disagreements happened, we were usually able to solve them. | ○ | ○ | ○ | ○ | ○ | ○ | ○ |
| We openly shared ideas with each other. | ○ | ○ | ○ | ○ | ○ | ○ | ○ |
| We often failed to communicate information to each other. | ○ | ○ | ○ | ○ | ○ | ○ | ○ |
| In general, it was difficult to converse with my partner. | ○ | ○ | ○ | ○ | ○ | ○ | ○ |

*Figure S5: Survey items to measure cooperative communication*

A perceived cooperative communication measure was calculated by calculating the average across the seven ratings (Items 2,3,6, and 7 are reverse scored).

# Analyses and Discussion

## Study 1

### Summary Statistics

Table S1: Frequency of responses per response category for perceived smart reply measure

| **Perceived Smart Reply Use** | **1 “Never”** | **2** | **3** | **4** | **5 “Always”** |
|---|---|---|---|---|---|
| **Frequence of responses per response category** | 132 | 115 | 58 | 36 | 20 |

Table S2: Means and standard deviations for study 1 variables.

| **Variable** | **Mean** | **Standard Deviation** |
|---|---|---|
| **Dominance** | -0.796 | 5.507 |
| **Affiliation** | 12.261 | 9.968 |
| **Cooperative Communication** | 37.989 | 8.399 |
| **Message Sentiment** | 0.094 | 0.135 |
| **Message Sentiment (no SR)** | 0.09 | 0.143 |
| **Communication Speed (msg/min)** | 1.621 | 0.674 |

### Potential Limitations

One potential limitation of our study design is that demand effects from using the perceived smart reply use measure impacted our findings. However, we have three reasons that support

our conclusions and add confidence to our reasoning that our findings about the impact of perceived smart reply use are not caused by demand effects.

First, all three measures (perceived smart reply use, cooperative communication and IAS-R) were randomized in order to control for potential order effects.

Second, while it is indeed plausible that our perceived smart reply use measure might have increased the saliency of thoughts about smart replies, we think it is unlikely to have impacted our findings. We think so because at the time we ran our study, smart replies were already common across many platforms and it is likely that people might have thought about their use regardless of the measure.

Finally, and most importantly, we believe that even if there were demand effects they would not weaken our findings and conclusions. It seems that demand effects, if present, could have only weakened our findings since they would reduce the impact of our manipulation. More specifically, a demand effect would mean that participants always thought about their partner using smart replies when answering our survey regardless of the condition they were in. As such it’s even more interesting that we find strong differences across conditions, despite the possibility that participants might have expected their partner to use them anyways.

# Study 2

## Summary Statistics

Table S3: Means and standard deviation for study 2 variables

| Variable | Mean | Standard Deviation |
|---|---|---|
| **Conversation Sentiment** | 0.112 | 0.135 |
| **Conversation Sentiment (no SR)** | 0.09 | 0.143 |
| **Conversation Affect** | 10.842 | 5.735 |
| **Conversation Affect (no SR)** | 10.583 | 5.408 |

# References

Knutson, B. (1996). Facial expressions of emotion influence interpersonal trait inferences. Journal of Nonverbal Behavior, 20(3), 165-182.

Lee, J. (1997). Leader-member exchange, the" Pelz Effect," and cooperative communication between group members. Management Communication Quarterly, 11(2), 266-287.

Wiggins, J. S., Trapnell, P. & Phillips, N. Psychometric and geometric characteristics of the Revised Interpersonal Adjective Scales (IAS-R). Multivar. Behav. Res. 23, 517–530 (1988).
